# Anatomical variants of pulmonary segments and uni-portal thoracoscopic segmentectomy for lung cancer in a patient with Kartagener syndrome: a case report

**DOI:** 10.1007/s11748-021-01685-3

**Published:** 2021-07-20

**Authors:** Di Zhou, Ye Tian, Yao Lu, Xueying Yang

**Affiliations:** Department of Thoracic Surgery, The Fourth Affiliated Hospital of China Medical University, No. 4 Chongshandong Road, Huanggu District, Shenyang, 110032 Liaoning Province China

**Keywords:** Kartagener syndrome, Situs inversus totalis, Anatomic segmentectomy, Lung cancer, VATS

## Abstract

Situs inversus totalis (SIT) is an extremely uncommon congenital disease where the major organs of the body are transposed through the sagittal plane. Kartagener syndrome is a complication of SIT with immotility of bronchial cilia, bronchiectasis, and chronic sinusitis. There is no report describing patients with Kartagener syndrome who accept uni-portal segmentectomies for lung cancer in past studies. Here we report a 74-year-old female patient with both Kartagener syndrome and a small early-stage lung cancer lesion located in the apical segment of the left upper lobe (LS^1^). The pulmonary segment anatomy of the left upper lobe in this case, which had very rare variants, was presented and interpreted in detail. This patient underwent an anatomic segmentectomy to the LS^1^ and a partial excision to the left middle lobe with bronchiectasis through a single 3 cm length incision. We believe that the case can give surgeons some experience and inspiration.

**Supplementary Information:**

The online version contains supplementary material available at 10.1007/s11748-021-01685-3.

## Introduction

Situs inversus totalis (SIT) is a rare congenital and autosomal recessive genetic disease which is connected with X-chromosome. It occurs in 1–2/10,000 newborns [1]. A complete reversal of the major organs’ normal arrangement within the thorax and abdomen is involved by SIT, which causes the patient with SIT to present inversed but proper anatomy [2]. In 20–25% of SIT cases, it coexists with Kartagener Syndrome (immotility of bronchial cilia, bronchiectasis, chronic sinusitis, besides SIT) [3]. Moreover, there are also more anatomical variants occurring in SIT than normal, especially the pulmonary variants that were the most frequently reported [4]. The enormous anatomical variation and uncomfortable reversal anatomy are the greatest challenges for thoracic surgeons. Here we describe the anatomical variants of pulmonary segments and uni-portal thoracoscopic segmentectomy for a lung cancer patient with Kartagener Syndrome.

## Case

**Fig. 1:**
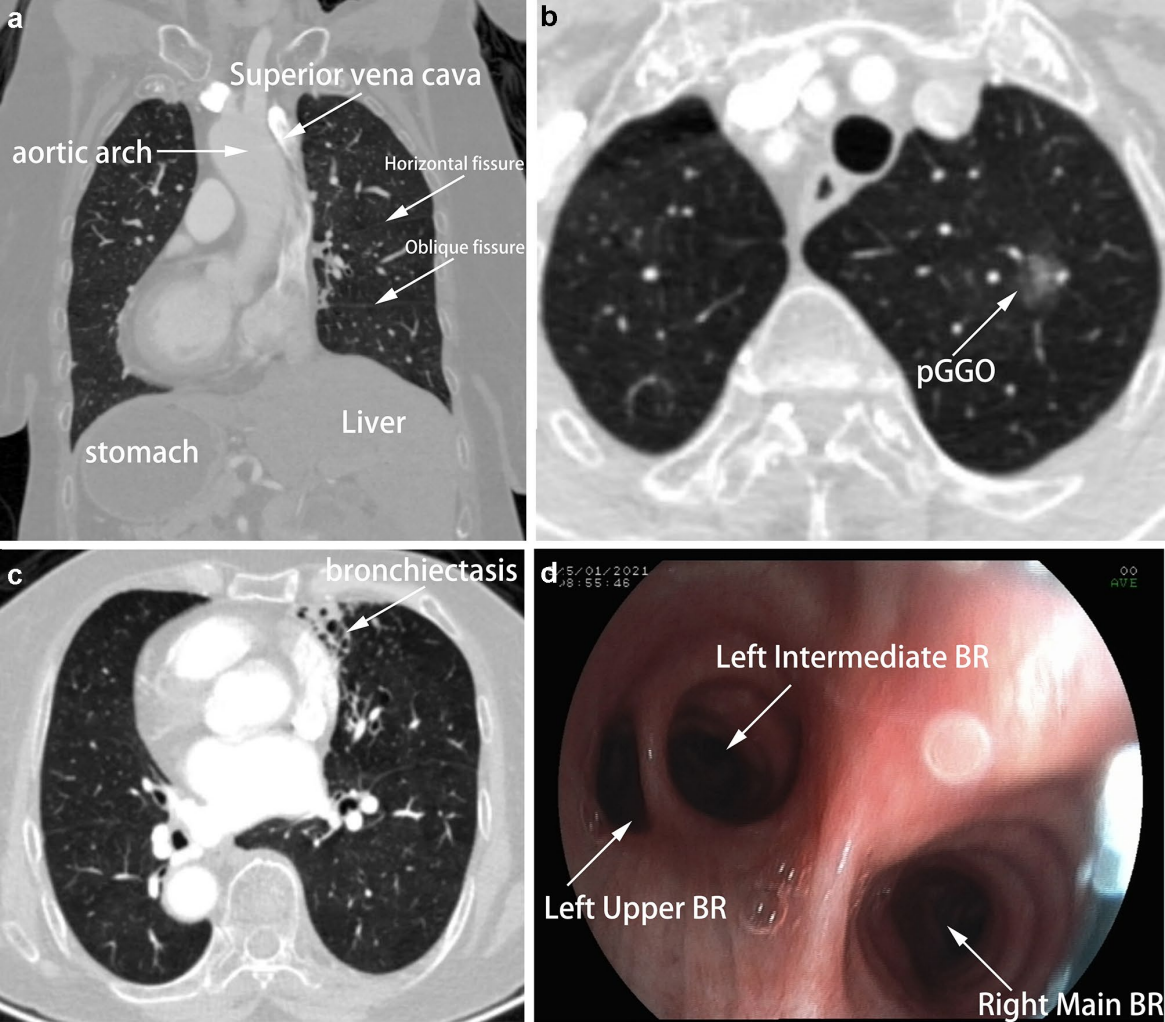
The radiological findings of the pulmonary tumor and thoracic anatomy. **a** Situs inversus totalis (dextrocardia, aortic arch, stomach, and liver). **b** Chest CT revealed a pure ground-glass opacity (pGGO) (measuring 10 × 12 mm in diameter) located in LS^1^. **c** Bronchiectasis with local inflammation was seen in the left middle lobe. **d** Bronchoscopy confirmed that the left and right bronchial branches were mirror-images of each other

A 74-year-old female non-smoker patient was hospitalized for an abnormal shadow found by computed tomography (CT) during her routine health checkup. She had no symptoms other than nasal congestion and sore throat, which were considered to be related to her history of chronic sinusitis. Chest CT revealed SIT (Fig. 1a) and a pure ground-glass opacity (pGGO) (measuring 10 × 12 mm in diameter) located in the apical segment (LS^1^) of the left upper lobe (Fig. 1b). Besides, bronchiectasis with local inflammation was seen in the left middle lobe (Fig. 1c). Bronchoscopy also confirmed that the left and right bronchial branches were mirror-images of each other (Fig. 1d). This patient had a preoperative diagnosis of pGGO in the left upper lobe and Kartagener Syndrome then was proposed for LS^1^ segmentectomy and left middle lobe partial resection under uni-portal video-assisted thoracoscopic surgery (VATS).

### Anatomical variants

**Fig. 2:**
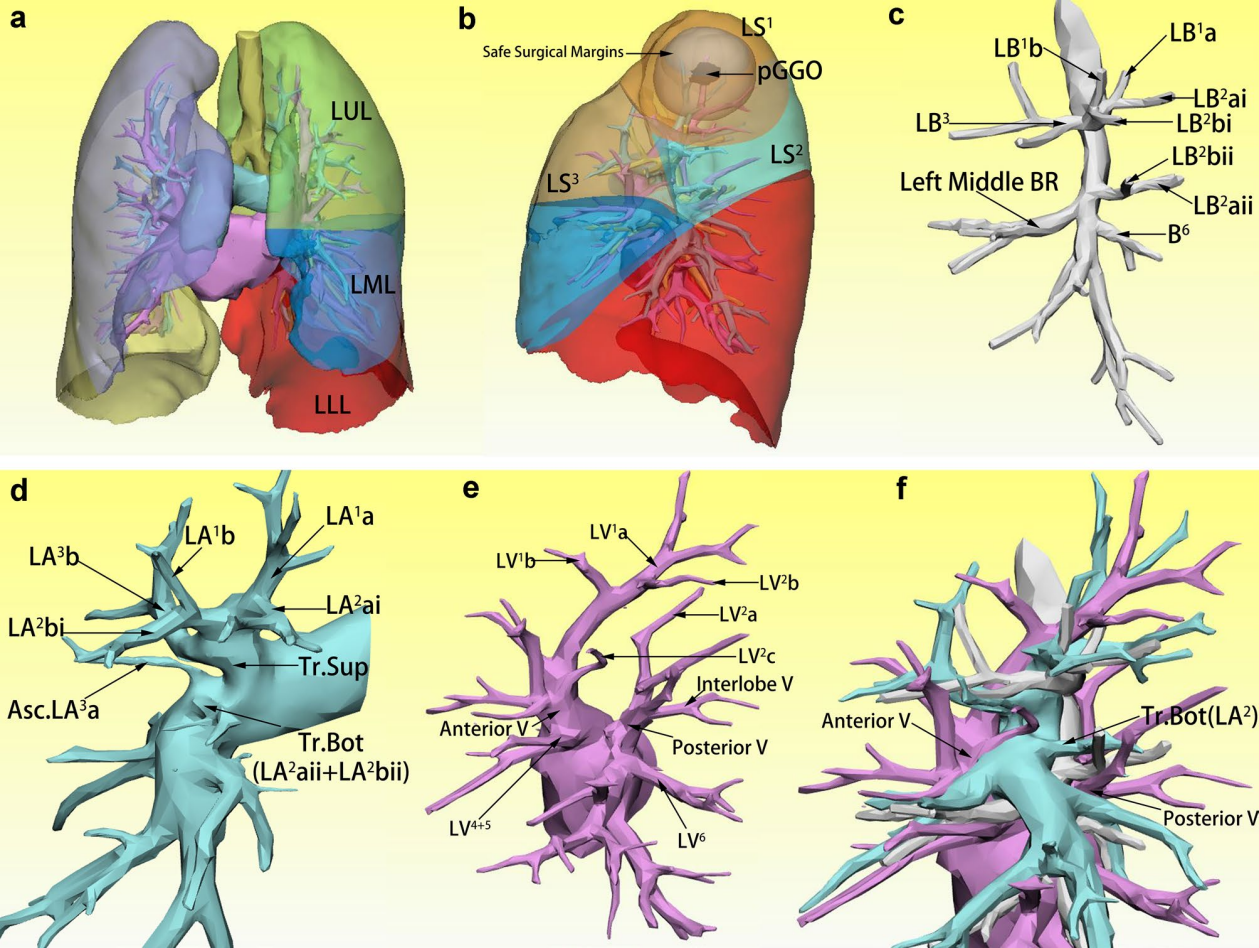
3D-CT images and designed surgical procedure. **a** left lung consisted of three lobes, the upper, middle, and lower lobes respectively. **b** The pGGO and the safe surgical margins were all completely within the LS^1^. **c** Anatomical variation of the left upper lobe bronchi. **d** Anatomical variation of the left upper lobe pulmonary arteries. **e** Anatomical variation of the left upper lobe pulmonary veins. **f** Key points of variation in the anatomical structure of the left upper lobe

Patients with SIT often have a combination of malformations or variants in other organs, and the variants of the pulmonary segment anatomy are also frequent, the combination of which makes three-dimensional CT (3D-CT) even more important to them for segmentectomy. To visualize the bronchi and pulmonary arteries precisely, we reconstructed 3D-CT images before the surgery (Fig. 2).

3D CT revealed a mirror reversal of the thoracic organs, with the left lung consisting of three lobes, the upper, middle, and lower lobes respectively (Fig. 2a). This pGGO smaller than 2 cm located in LS^1^ and the safe surgical margins (its outward extension of 2 cm) was also completely within the LS^1^(Fig. 2b), which proved that segmentectomy is completely feasible.

The bronchi of the left upper lobe contained two main trunks. The upper trunk was divided into two branches, among them, LB^3^ emanated alone, LB^1^b and LB^1^a merged with LB^2^bi and LB^2^ai respectively to form a branch resembling an antler. In addition, there is another trunk, which contained LB^2^bii and LB^2^aii, originating above B6, which is basically at the same level as the left middle bronchi (Fig. 2c).

Variation of the pulmonary arteries in the left upper lobe occurs in conjunction with the variation of the bronchi, because of the proven concomitant relations between them. In the study of Nagashima et al. [5], the pulmonary arteries in the right upper lobe were defined as Tr.sup, Tr.inf, and Asc.A. But in this case, pulmonary arteries in the left upper lobe, which is the mirror image of the convention, had a trunk that cannot be classified in the above way. Although emanating from a location roughly of Tr. Inf, this artery was the third branch of the left main pulmonary artery, later than Asc.A^3^a. So, we defined it as trunk bottom (Tr. Bot), which emanates from the pulmonary artery stem with the level of the upper lobe bottom and below the ascending artery, finally travels to the posterior and/or anterior segment. In this case, the pulmonary arteries in the left upper lobe were composed of Tr. Sup, Asc.A^3^a, and Tr. Bot. Similar to the bronchi, LA^1^b and LA^1^a merged with LA^2^bi and LA^2^ai respectively, and then together with A^3^b formed the Tr. Sup. Tr. Bot contained A^2^aii and A^2^bii, then covered the lower half of the posterior segment in the left upper lobe (LS^2^) (Fig. 2d).

In the study by Shimizu et al. [6], pulmonary veins of the right upper lobe were divided into anterior V and central V according to their relationship to the bronchi. Veins traveling between B^2^ and B^3^ were defined as central V while traveling anterior to the right upper lobe bronchus were defined as anterior V. However, there was a trunk of veins that traveled posteriorly to the left upper lobe bronchus in this case, and we defined it as posterior V. The anterior V composed of V^1^, V^3^, V^2^b, and V^2^c, meanwhile the posterior V composed of V^2^a and interlobe V (traveled in an undifferentiated posterior oblique fissure between left upper and lower lobe). There was no central V in this case, Anterior V and posterior V were jointly responsible for venous reflux of the left upper lobe (Fig. 2e and f).

### Surgical treatment

A uni-portal thoracoscopic segmentectomy of the LS^1^ and left middle lobe partial resection were conducted according to the preoperative 3D-CT and designed surgical procedure.

**Fig. 3:**
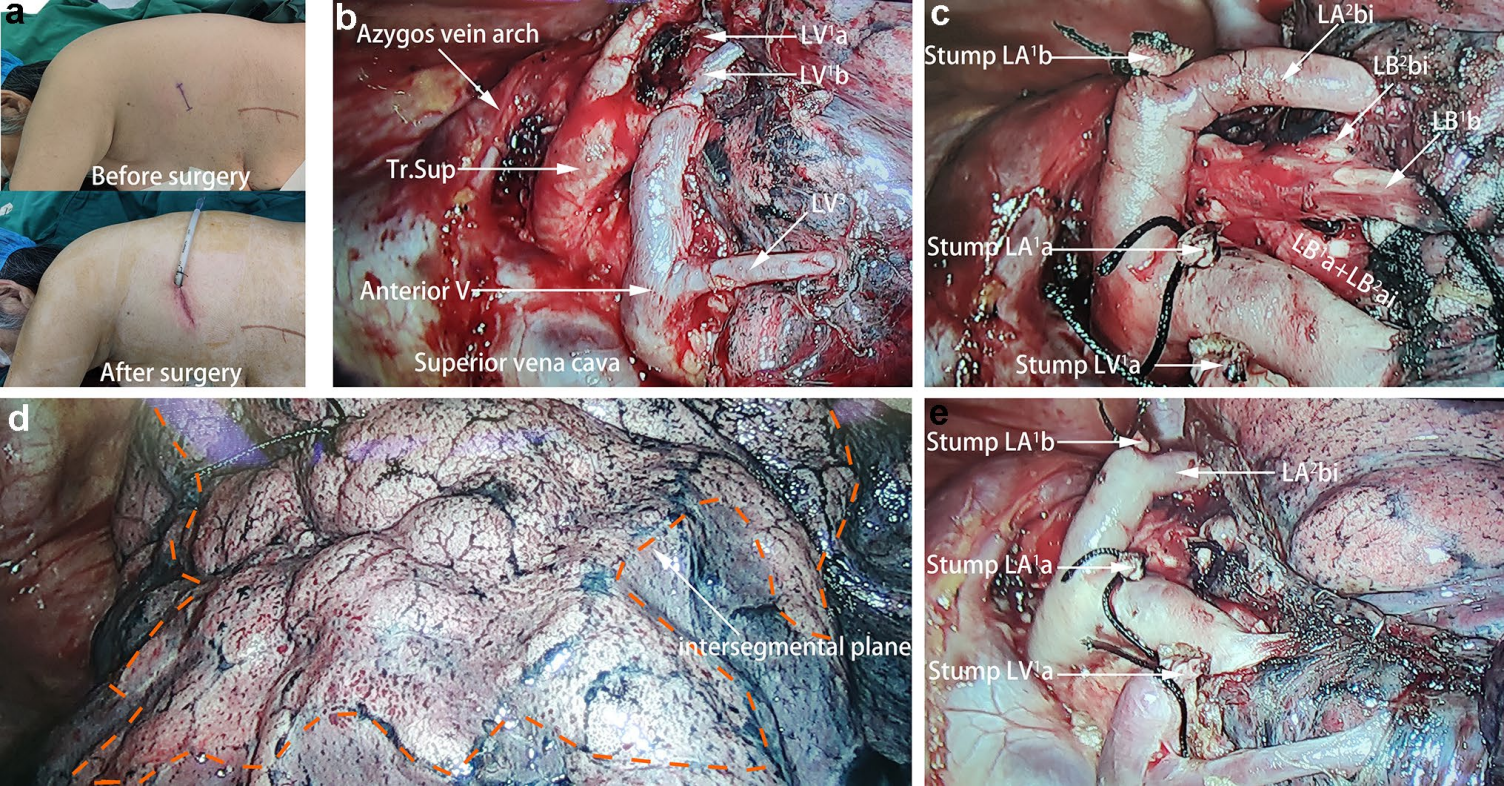
The anatomic segmentectomy by uni-portal approach. **a** Only a single 3 cm incision for the surgery. **b** Azygos vein arch and superior vena cava were mirror images of a normal condition. The anterior mediastinal pleura is disassociated to expose the pulmonary artery and vein roots. **c** The target pulmonary arteries and veins were cut off. **d** intersegmental plane after the inflation-deflation technique. **e** Anatomy of segmental hilum after LS^1^ resection

The patient was placed in a right lateral decubitus position. A VATS approach through a single 3 cm incision was made in the left 5th intercostal space (Fig. 3a). The left lung composition of three lobes, vascular and bronchial anatomy was exactly like a normal right lung. The aortic arch and descending aorta were not shown. Azygos vein arch and superior vena cava were mirror images of a normal condition (Fig. 3b). A few adhesions exist between the left middle lobe and the anterior chest wall and pericardium, which can be easily broken with an electric hook.

The mediastinal pleura was incised above the anterior hilum of the lung while the pulmonary artery and vein root of the left upper lobe was exposed (Fig. 3b). The 11 and 12 lymph nodes affecting our operation were removed and pathologically examined, with the final result being no malignant tumor tissue. We dissociated along with the left upper pulmonary vein distally to expose their various branches, including V^3^, V^2^c, V^1^b, V^2^b, and V^1^a in sequence, then cut off V^1^a. After this, we dissociated along with the Tr. Sup of the left pulmonary artery, and exposed A^3^b, A^2^bi, A^1^b, A^2^ai, and A^1^a sequentially. A^1^b and A^1^a were cut off afterward. With the target segment artery severed, B^1^a and B^1^b, which traveled on the deeper side of A^1^a and A^1^b, also became easily exposed and cut off (Fig. 3c). The identification of the intersegmental plane was defined after the inflation-deflation technique (Fig. 3d), and then we removed LS^1^ by a linear stapling cutter (Fig. 3e). Additionally, we performed a partial resection of the left middle lobe to remove the bronchiectasis lesion. The final pathological examination revealed adenocarcinoma in situ (AIS) in LS^1^, while bronchiectasis and inflammatory lesions with neuroendocrine cell nodular hyperplasia in the left middle lobe. Considering the low incidence of lymph node metastasis in adenocarcinoma in situ, with the consent of the patient's family, no lymph nodes were dissected except for the 11 and 12 resected intraoperatively.

The total surgical time was 82 min. The patient had hypoxemia for a time due to poor postoperative sputum elimination, which was thought to be possibly related to the immotility of bronchial cilia in Kartagener Syndrome, but the symptom was quickly relieved by a single bedside fiberoptic bronchoscopic aspiration and did not recur. The chest tube was removed on the second postoperative day and the patient was discharged on the sixth postoperative day.

## Discussion

SIT is an uncommon congenital disease in which the organs of the body are transposed through the sagittal plane. Kartagener syndrome is a complication of SIT with immotility of bronchial cilia, bronchiectasis, and chronic sinusitis. Most published reports about surgery for SIT patients are abdominal procedures and the experience in VATS is limited. The first case report of SIT in thoracoscopic lobectomy was published in 2013 [4], since then few cases on thoracoscopic lobectomy in patients with SIT have published, and cases on thoracoscopic segmentectomy are even fewer. Most of them are by multi-portal approach. Diego Gonzalez-Rivas published the first and only report of anatomic segmentectomy by the uni-portal approach in a patient with SIT [7]. The uni-portal approach causes less trauma and pain than the multi-portal approach but requires more skill and patience on the part of both the operator and assistant. Especially due to the relative fixation of the camera in the uni-portal approach, the discomfort caused by the mirror-image in SIT patients is increased. But overall, the difficulty of uni-portal in patients with SIT is not fundamentally different from the common uni-portal thoracoscopic surgery. Surgery for cancer is much rare than for bronchiectasis to patients with Kartagener syndrome, and some scholars reported that due to chronic and repeated infections, intrathoracic adhesions and expanded bronchial artery had made the operation more complicated [8]. However, in our case, irregular and expanded bronchial arteries were not found, and only a few intrathoracic adhesions need to be branded off, which did not affect the operation. This may be related to the milder condition of bronchiectasis in this case, suggesting that the progression of bronchiectasis in Kartagener syndrome also requires a process, the length of which may vary from person to person. In addition, postoperative hypoxemia in our case may be due to the immotility of bronchial cilia, which needs to be taken seriously. In the experience of this single Kartagener syndrome patient, perioperative airway management is very important. Glucocorticoid nebulized inhalation and the use of sputum-reducing drugs are very effective, while early fiberoptic bronchoscopic aspiration is essential to avoid complications such as hypoxemia and pulmonary atelectasis once the patient fails to cough up sputum effectively. To our knowledge, this is the first report of uni-portal thoracoscopic anatomic segmentectomy in a patient with Kartagener syndrome.

Thanks to the increased popularity of health checkups and advances in imaging techniques, the detection of early-stage of lung cancer is growing, especially the GGO lesions. An anatomic segmentectomy could bring a satisfactory prognosis to patients with GGO smaller than 2 cm, both to secure an adequate surgical margin, remove distal peri-bronchial lymph nodes, and preserve pulmonary function [9, 10]. However, the anatomical structure of the pulmonary segment is complex and varied, it is difficult to pinpoint pulmonary nodules, identify surgical margins, detect anatomical variations and protect intersegmental veins [11]. Especially in cases with SIT, not only are the organs reversed which will bring an uncomfortable and unaccustomed feeling to surgeons but there may also be more variations in the anatomical structure of the pulmonary vessels and bronchi. Therefore, the 3D-CT, which can give surgeons a more visual and accurate presentation of the pulmonary vessels and bronchi, is useful to understand the mirror-imaged anatomy and to prevent the risk of vascular and bronchial injury.

## Conclusion

Though we complete the operation smoothly, Kartagener syndrome still is a challenge to surgeons, especially when performing uni-portal VATS anatomic segmentectomy, which needs not only precise 3D-CT and planning before the surgery but also skilled Uniportal VATS surgeons to perform.

## Supplementary Information

Below is the link to the electronic supplementary material.

Video 1. Anatomical Variants of Pulmonary Segments and Uni-portal Thoracoscopic Segmentectomy for Lung Cancer in a patient with Kartagener SyndromeVideo 1. Anatomical Variants of Pulmonary Segments and Uni-portal Thoracoscopic Segmentectomy for Lung Cancer in a patient with Kartagener Syndrome (MP4 55955 kb)

Supplementary Video 1. The CT image of this case (MP4 257563 kb)
